# Tricuspid valve infective endocarditis in a patient with psoas abscess complicated by septic pulmonary emboli and severe tricuspid regurgitation in Cameroon: challenges in the diagnosis and management in a resource limited setting (a case report)

**DOI:** 10.11604/pamj.2022.41.300.33057

**Published:** 2022-04-14

**Authors:** Clovis Nkoke, Jerry Brown Aseneh, Emmanuel Njang, Conrald Metuge Ekukole, Kingsly Nkongho Enoh

**Affiliations:** 1 Buea Regional Hospital, Buea, Cameroon,; 2 Health Education and Research Organization (HERO), Buea, Cameroon,; 3 Solidarity Hospital, Buea, Cameroon

**Keywords:** Laminectomy, psoas abscess, tricuspid infective endocarditis, case report

## Abstract

Infective endocarditis of the tricuspid valve is rare in non-intra-venous drug abusers. Few cases of psoas abscess complicated by tricuspid infective endocarditis have been reported. A 61-year-old man underwent a laminectomy. Three weeks later he developed persistent fever, abdominal pain, back pain and hip pain, weight loss, gradually and abdominal distension. Abdomino-thoracic computed tomographic scan showed a left psoas muscle abscess and cavitary pulmonary lesions suggestive of septic pulmonary emboli. Two dimensional transthoracic echocardiography showed an oscillating mass on the anterior leaflet of the tricuspid valve compatible with a vegetation. There was severe tricuspid regurgitation with right atrial and right ventricular dilatation. Secondary psoas abscess though rare is an important cause of bacteremia and there is a potential of bacteremia progressing to serious systemic infection like tricuspid endocarditis which can be fatal without prompt and appropriate treatment.

## Introduction

Lumbar laminectomy a common lumbar procedure for spinal stenosis [1]. It can result in several complications, one of which is the rare occurrence of a psoas abscess with systemic infection resulting in tricuspid infective endocarditis. Right sided infective endocarditis mostly commonly occurs in intravenous drug abusers [2]. Other risk factors for right sided infective endocarditis include presence of a cardiac implantable electronic device (CIED) or other intravascular device, and presence of an underlying right-sided cardiac anomaly [2]. Psoas abscess is a rare complication of laminectomy that can potentially lead to significant systemic infection. We report a case of psoas abscess associated with tricuspid valve endocarditis in a 61-year-old patient who underwent laminectomy indicated for lumbar spinal stenosis.

## Patient and observation

**Patient information:** a 61-year-old patient with no significant past history underwent L4-L5 laminectomy indicated for lumbar canal stenosis. Following the intervention, he requested to be discharged and continue wound dressing on out-patient basis. Three weeks later, he developed a constant low-grade fever, associated with left flank pain, hip pain and back pain.

**Clinical finding:** on physical examination, the patient looked cachectic with a tender distended abdomen and bi-pedal edema. His temperature was 38.9°C. On cardiovascular examination there were distended neck veins, hepatojugular reflux and a grade 4 pansystolic murmur of tricuspid regurgitation. The lung examination was normal. The wound dressing on his back was soiled and oozed a purulent discharge.

**Figure 1:**
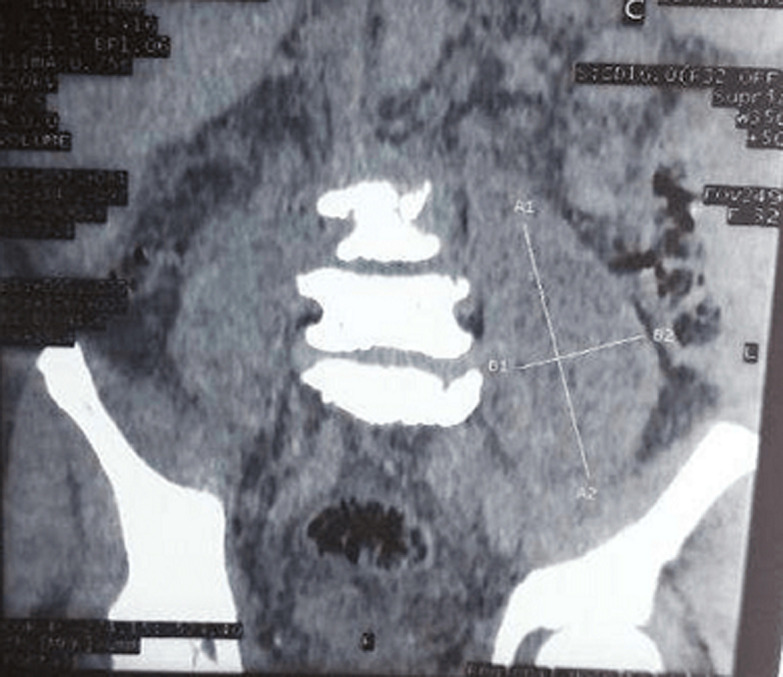
left psoas abscess on abdominal CT scan

**Figure 2:**
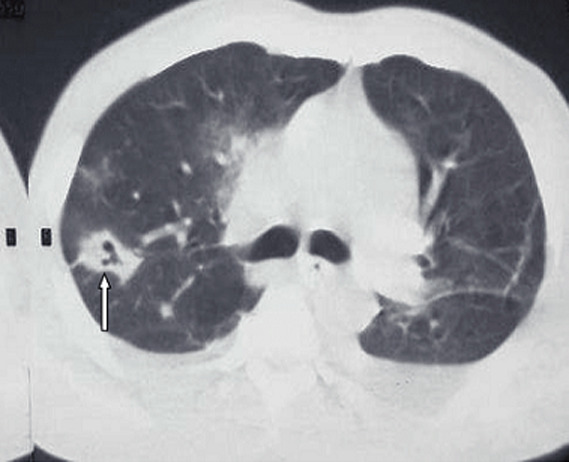
septic pulmonary embolus (arrow) chest CT scan

**Figure 3:**
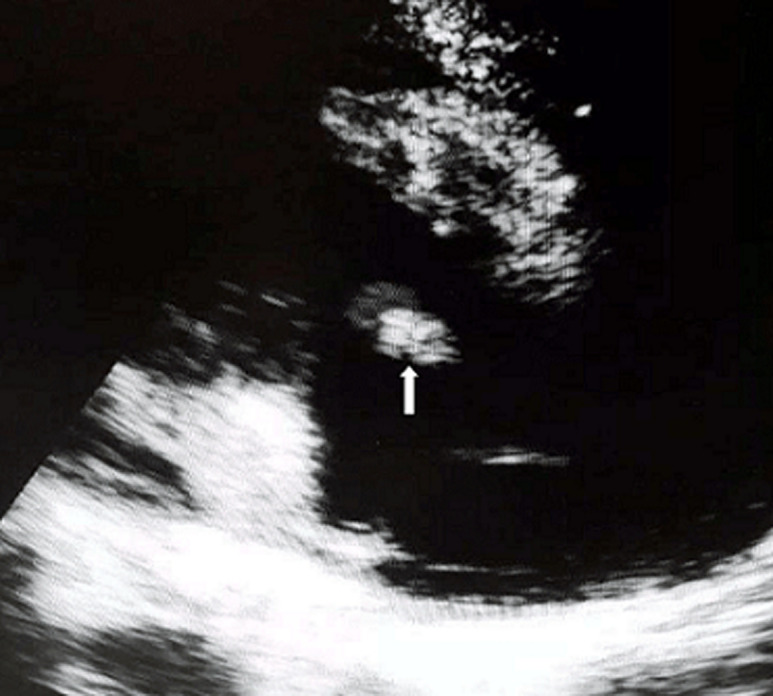
transthoracic echocardiography showing vegetation on anterior leaflet of tricuspid valve (white arrow) on apical four chamber view

**Figure 4:**
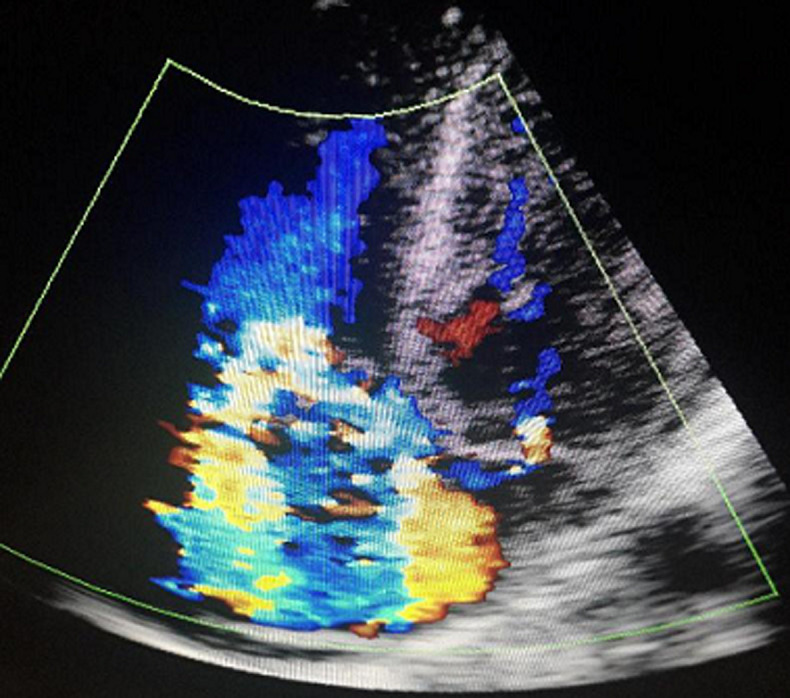
transthoracic echocardiography showing severe tricuspid regurgitation on apical four chamber view

**Diagnostic assessment:** a thoraco-abdominal computed tomographic scan done following this revealed a left psoas abscess with dimensions of 90 x 42 x 40mm (Figure 1). In the lower chest there were thick walled cavitary lesions suggestive of septic pulmonary emboli (Figure 2). Full blood count showed hyperleukocytosis at 31,900 /mm^3^. The hemoglobin was 8.2g/dl. Two dimensional echocardiography performed to look for a right sided infective endocarditis because of the septic pulmonary emboli on the CT scan. It showed the presence of an oscillating mass on the anterior leaflet of the tricuspid measuring 13mm x 7mm, compatible with a vegetation (Figure 3). There was dilatation of the right ventricle and the right atrium with severe tricuspid regurgitation (Figure 4). The diagnosis of infective endocarditis with septic embolization to the lungs was made.

**Therapeutic intervention:** the right heart failure was treated with intravenous diuretics and the patient was put on intravenous oxacillin 4g three times daily, gentamycin 1mg per kilogram daily and ceftriaxone 1g twice daily for the treatment of infective endocarditis. The empiric antibiotherapy was to target staphylococcus and streptococcus. Due to limitations in the setting, blood cultures were not performed. The psoas abscess was drained percutaneously.

**Follow-up and outcome:** the patient died 2 days after the diagnosis of infective endocarditis and after instituting empirical anti-biotherapy.

**Ethics approval and consent to participate:** written informed consent was obtained from the patient for publication of this case report and any accompanying images.

## Discussion

Infective endocarditis is a relatively rare yet serious and potentially fatal infection. Infective endocarditis most commonly involves the mitral and aortic valves. Right-sided infective endocarditis is rare. It represents 5-10% of all infective endocarditis cases [2]. It most commonly affects patients with a history of intravenous drug abuse; however, it is also associated with patients on dialysis, or patients who have intra-cardiac devices, congenital heart diseases or immunocompromised states [2].

Psoas abscess is a rare clinical entity and it is difficult to diagnose due to its insidious onset and nonspecific symptoms. A psoas abscess can be secondary or primary. Primary psoas abscess originates from an infection of distant source spread through hematological or lymphatic routes. Secondary psoas abscess is an infectious process involving adjacent structures via direct invasion. Conditions associated with secondary psoas abscess include Crohn´s disease, diverticulitis, appendicitis, colorectal cancer, urinary tract infection, vertebral osteomyelitis, mycotic abdominal aortic aneurysm, endocarditis, and history of instrumentation in or around the spine [3,4]. The patient presented in this case report had a laminectomy 3 weeks prior. Thus, there was instrumentation around the spine. This was the most probable cause of the psoas abscess. Bloodstream infection is a prerequisite for development of native valve infective endocarditis. Septic pulmonary emboli are indicative of bacteremia as it was the case in our patient. It is most likely that the psoas abscess later caused a bacteremia with colonization of the tricuspid valve causing infective endocarditis. It is rare for an abscess to cause infective endocarditis. There have been sporadic reports of Staphylococcus bacteremia complicated by psoas abscess and infective endocarditis but it was the mitral valve that was affected [5]. In that report, the patient had atopic dermatitis. Patients with atopic dermatitis are more vulnerable to infection due to impaired skin barrier. *Staphylococcus aureus*, in particular, is frequently seen in atopic dermatitis patients´ skin lesions [6]. Instrumentation in or around the spine is a secondary cause of psoas abscess [3,4]. In our patient the mostly likely source of the bacteremia that caused the tricuspid endocarditis was the psoas abscess which resulted from the laminectomy indicated for lumbar spinal stenosis.

Psoas abscess has been reported to be associated with cardiovascular disease [7]. In one report of psoas abscess associated with cardiovascular disease, 26.6% of the 15 patients had infective endocarditis [7]. The mortality is higher for secondary psoas abscess than for primary psoas abscess, especially those associated with cardiovascular disease. The mortality reaches up to 100% if the condition is left untreated [8]. Our patient received empirical antibiotic therapy to target staphylococcal species. *Staphylococcus aureus* is the most common microorganism in both primary and secondary psoas abscess related to skeletal muscular infections [4,9,10]. It was not possible to do blood cultures in our patient to identify the germ at the facility. This poses difficulties and challenges in identifying the germ and instituting appropriated anti-biotherapy based on sensitivity studies. *Staphylococcus aureus* is a very aggressive pathogen and bacteremia from this germ can infect healthy heart valves. Also, our patient had severe tricuspid regurgitation which required surgical treatment or transcatheter tricuspid valve intervention (TTVI) but this was not possible in our setting due to limitations [11]. Antibiotic therapy and surgical options remains a mainstay of successful treatment of tricuspid valve infective endocarditis. Surgical treatment is indicated tricuspid valve vegetation > 2cm with septic pulmonary emboli, persistent bacteremia for one week despite adequate treatment, and severe tricuspid regurgitation with right-sided heart failure [12]. Our patient had two indications for surgical treatment. But could not be performed because of no access to emergency surgical care due to limited cardiac surgical centers and financial constraints.

## Conclusion

Secondary psoas abscess though rare is an important cause of bacteremia and there is a potential of bacteremia progressing to serious systemic bacterial infection such as infective endocarditis which can be fatal. This poses a diagnostic and therapeutic challenge in resource limited settings like ours.
